# Wealth inequalities in physical and cognitive impairments across Japan and Europe: the role of health expenditure and infrastructure

**DOI:** 10.1186/s12939-023-01906-6

**Published:** 2023-06-29

**Authors:** Dung D. Le, Nekehia T. Quashie, Martina Brandt, Yoko Ibuka

**Affiliations:** 1 Faculty of Economics, Keio University, 2-15-45 Mita, Minato-Ku, Tokyo 108-8345 Japan; 2 Department of Health Studies, University of Rhode Island, Kingston, RI 02881 USA; 3 Faculty of Social Sciences, TU Dortmund, Emil Figge Str. 50, 44227 Dortmund, Germany

**Keywords:** Health inequality, Physical and cognitive impairments, Wealth inequality, Welfare state, Expenditure approach, Cross-country comparisons, Japan, Europe

## Abstract

Although prior research has provided insights into the association between country-level factors and health inequalities, key research gaps remain. First, most previous studies examine subjective rather than objective health measures. Second, the wealth dimension in health inequalities is understudied. Third, a handful of studies explicitly focus on older adults. To bridge these research gaps, this study measures wealth-related inequalities in physical and cognitive impairments and examines the extent to which welfare states moderate wealth inequalities in physical and cognitive impairments among older people across Japan and Europe. We utilized harmonized data on non-institutionalized individuals aged 50–75 from the Japanese Study of Aging and Retirement (JSTAR) and the Survey of Health, Ageing and Retirement in Europe (SHARE) (*N* = 31,969 for physical impairments and 31,348 for cognitive impairments). Our multilevel linear regression analyses examined whether national public health spending and healthcare access resources explained cross-country differences in wealth inequalities in physical and cognitive impairments. We applied a concentration index to quantify the degree of wealth inequalities in impairments. The findings indicate that inequalities in both impairment outcomes favored wealthier individuals in all countries, but the magnitude of inequality varied by country. Furthermore, a higher share of public health spending, lower out-of-pocket expenditure, and higher investment in healthcare resources were associated with lower wealth inequalities, especially for physical impairments. Our findings suggest that different health interventions and policies may be needed to mitigate specific impairment inequalities.

**Supplementary Information:**

The online version contains supplementary material available at 10.1186/s12939-023-01906-6.

## Introduction

The socioeconomic gradient in health across the life span is well established [1–6] and the magnitude of socioeconomic health inequalities varies across countries [7–9]. To consider how to address socioeconomic health disparities, research suggests that welfare state structures are a key determinant due to varying social policies (e.g., education expansion, healthcare access, and benefits for vulnerable groups such as the unemployed and disabled), which may explain how individual socioeconomic (dis)advantage translates into health inequalities [10–12]. Nevertheless, empirical findings on the association between welfare state structures and health inequalities vary depending on whether the regime, institutional, or expenditure approach is applied [10, 11]. Given global variations in the existence of welfare organizations to support vulnerable groups as well as the growing share of older adults likely to rely on welfare provisions such as social protection and healthcare, there is a pressing need for refined assessments of the effect of the welfare state on health inequalities in aging societies. Japan and European countries are among the global frontrunners of population aging with adults 65 years and older accounting for 20% or more of the respective populations [13]. Yet, there is substantial diversity of social welfare systems across European countries and Japan, offering compelling conditions to examine the extent to which welfare states might moderate socioeconomic inequalities in the health of older persons.

Despite having increased health risks and healthcare needs than younger individuals, older people tend to have more difficulties in accessing affordable healthcare, potentially generating inequalities in health and healthcare access [14]. Additionally, cross-national studies suggest that populations tend to be healthier in countries with higher wealth, generous social welfare expenditure, and low income inequality [8, 15]. Some studies, however, show that health disparities are more pronounced in wealthier countries [9] or unrelated to the distribution of resources within a country [15]. Although some advanced aging societies in Europe (e.g., Czech Republic, Spain, and the United Kingdom) have managed to balance low public spending on healthcare with maintaining relatively good healthcare access [16], it remains unclear whether higher levels of public health spending and healthcare resources mitigate health inequalities among older adults.

While previous studies provide important insights into the association between welfare states and health inequalities, four research gaps are evident. *First*, there has been extensive use of subjective health outcomes, namely, self-rated health (SRH) [17–20], limiting our understanding of other health dimensions such as cognitive impairments, which are increasingly prevalent and require intense caregiving resources [21, 22]. Our study examines both physical (e.g., self-reports of functional impairment/disability) and cognitive impairments (e.g., memory tests conducted by interviewers). The former are considered as a quasi-objective indicator of health, which arguably provides a more accurate assessment than SRH [23] and is likely less sensitive to the cross-cultural biases identified for SRH [24–26]. By contrast, the latter are objective measures of health that are less subject to individual recall bias. *Second*, a handful of studies have explicitly focused on older adults [15, 18, 27].

*Third*, wealth as an indicator of socioeconomic status (SES) is underexplored [15], as research has focused on income- or educational-related health inequalities [7, 19]. Household wealth, arguably a crucial indicator of later life SES, accounts for the accumulation of economic resources throughout the life course and serves as a better indicator of financial security to promote health and wellbeing in later life [28–30]. Furthermore, physical, and cognitive impairments are closely related to long-term care needs and accumulated wealth may be used to access formal care if co-payment is required since income tends to reduce in old age, especially after retirement. In the case of informal care, wealth may incentivize intergenerational transfers through inheritance in return for caregiving [31, 32]. *Finally*, although cross-national studies examining the association between health inequalities and macro determinants have exclusively been conducted in Western countries [17–20], less attention has paid to the roles of healthcare financing and infrastructure macro indicators, which are closely related to both the demand and the supply sides and may affect healthcare access. For example, healthcare access of the disadvantaged may be restricted if out-of-pocket (OOP) payments (demand side) are high and healthcare resources (supply side) are insufficient.

Our study bridges these gaps and contributes to comparative research on the socioeconomic health gradient in two ways. First, we measure wealth-related inequalities in physical and cognitive impairments among individuals aged 50 to 75 across 16 countries using harmonized data from the Survey of Health Ageing and Retirement in Europe (SHARE) and the Japanese Study of Aging and Retirement (JSTAR). Second, we examine the extent to which welfare state expenditure moderates wealth inequalities in physical and cognitive impairments.

## Background

Three approaches are commonly used to investigate the association between welfare state (macro) determinants and health inequalities: the regime, institutional, and expenditure approaches [10]. As these approaches intersect, it is difficult to empirically assess their underlying mechanisms independently in one study. The regime approach, commonly used in cross-national studies on health inequalities, argues that a given group of countries can be categorized according to analogous ideologies and policies or political traditions that inform population health (in)equity [19]. Despite a growing number of studies utilizing this approach to examine the association between health inequalities and macro regions [17, 33], the definition and application of typologies remain unclear [10]. The regime categories proposed by Esping-Andersen (liberal, conservative, and social democratic regimes) [34] and Ferrera (Anglo-Saxon, Bismarckian, Scandinavian, and Southern regimes) [35] are the most commonly used in cross-national studies. Empirical support for the regime approach is inconsistent, thereby limiting generalizable conclusions about the association between health inequalities and macro determinants [10, 20]. Moreover, countries may be misclassified, as clustering by broad welfare typology masks similarities in welfare policies (e.g., healthcare) across regimes [19, 36].

The institutional approach adopts a social rights-based approach, namely, social policies and programs including pension, sickness insurance, unemployment benefits, and family policies and their links to population health. The empirical findings using this approach consistently indicate that higher welfare state expenditure and benefits are associated with lower health inequalities [10]. A major drawback of this approach in cross-national analysis is that several assumptions are required to compute the social rights indices. Unemployment replacement rates, for example, are assumed for a “standard worker” who works in the manufacturing industry, is 30 years of age, worked for ten years and for 5 years at the current employer. Such assumptions may be problematic, as they are only applicable to a specific group of people [19, 37].

Finally, the expenditure approach exploits information on public spending on social purposes such as healthcare spending to measure welfare state effort and the degree of generosity. Similar to the institutional approach, the empirical findings are consistent: higher public social spending is associated with lower health inequalities and better overall population health [10]. However, this approach has been criticized for its difficulty in assessing the “true” meaning of welfare state generosity [19]. For example, in countries where social vulnerabilities such as unemployment are widespread, high public expenditure to address the issue does not capture the generosity of public policies but rather the large number of dependent recipients [10]. Yet, we consider that higher social expenditure to address social challenges of a large number of dependent persons in a society serves to improve the welfare of the most disadvantaged thereby contributing to reduced health inequality.

Although the existing empirical evidence specific to older adults is limited, studies suggest that the institutional and expenditure approaches provide more generalizable conclusions about the association between macro determinants and health inequalities than the regime approach. For instance, Högberg et al. (2018), using data from the European Social Survey, applied both the expenditure and the institutional approaches to examine the relationship between social class inequalities in SRH and limiting long-standing illness and macro indicators across 21 European countries [27]. The findings demonstrated that a higher minimum pension replacement rate and higher expenditure on older adult care are significantly associated with lower health inequalities in both SRH and limiting long-standing illness among those aged 65–80. Similarly, de Breij and colleagues (2020), using SHARE and the English Longitudinal Study of Ageing, investigated the link between several macro indicators classified by the expenditure and institutional approaches and educational health inequalities among retirees aged 50–65 in 18 European countries [18]. Their findings showed that higher social expenditure (including expenditure on health, old age, survivors, incapacity, family support, unemployment, and housing) is associated with better health and that higher old age, unemployment, health expenditure, and replacement rates are associated with lower health inequalities.

Given that the assumptions of the institutional approach are restrictive and not necessarily appropriate for older populations [18], our study adopts and expands on the expenditure approach by focusing on the relative importance of healthcare financing (e.g., public health expenditure and individual OOP expenditure) and healthcare infrastructure (number of doctors and hospital beds) on health inequalities among older adults in Europe and Japan. Healthcare financing schemes in European countries and Japan consist of four components: public spending, OOP payments, compulsory health insurance, and voluntary health insurance schemes [16]. Our study focuses on the first two major components. Public spending on healthcare varies considerably across countries, leading to differences in individuals’ financial responsibility for healthcare (i.e., OOP spending) and in the availability of healthcare facilities and services. Countries such as Greece, Spain, and Italy have even reduced public spending on healthcare due to financial crises [16].

High OOP expenditure as well as the insufficient availability of healthcare facilities and practitioners are major barriers to healthcare access in several European countries [16] and in Japan [38]. Socioeconomically disadvantaged individuals experience more inadequate access to healthcare and tend to live in areas characterized by a shortage of physicians and timely medical assistance [16, 39]. Structural and administrative challenges such as long waiting times are among the main reason for unmet medical check-ups in many European countries including Czech Republic, Denmark, the Netherlands, and Poland, which also tends to disproportionately affect financially disadvantaged groups [16]. Additionally, distance to medical institutions and geographical inequalities in healthcare provision have been highlighted as important reasons for unmet healthcare needs, and these are more pronounced in Central and Eastern European countries than in Nordic ones [16]. Overall, cross-national differences in healthcare financing and access to health resources suggest that welfare state health-related policies may mitigate or exacerbate individual socioeconomic health inequalities. However, studies have thus far failed to examine the effect of healthcare expenditure and infrastructure on socioeconomic health inequalities among older adults simultaneously.

The social resource perspective suggests that higher SES individuals have several advantages over those with a lower SES, such as better access to medical services, higher treatment quality, better preventive care, and greater access to healthy nutrition [40, 41]. Hence,

- *Hypothesis 1 (wealth-related inequalities in health): the prevalence of physical and cognitive impairments is concentrated among the less wealthy than wealthier individuals across the countries in this study.*

Aligned with the theoretical overview, we expect that generous public health spending and higher investment in healthcare resources, which tend to target socially disadvantaged groups, are more beneficial to the disadvantaged in the sense that less wealthy individuals, on average, may experience more health benefits (or improvements) than wealthier individuals, thereby potentially narrowing the gap in health inequality. Hence,

- *Hypothesis 2a (public healthcare financing): a higher share of public health spending is associated with smaller health inequalities in physical and cognitive impairments.*
- *Hypothesis 2b (private healthcare financing): a lower share of OOP health spending is associated with lower health inequalities in both impairments.*
- *Hypothesis 3 (healthcare access): a higher availability of doctors and hospital beds is associated with lower health inequalities in both impairments.*

## Methods

### Data

We used two harmonized datasets provided by the Gateway to Global Aging Data repository, which facilitates cross-national analyses by providing a range of metadata including questionnaires and the item comparability [42]. We combined cross-sectional data for comparable years from JSTAR (wave 1, 2007) and SHARE (wave 2, 2006–2007),^1^ yielding 16 countries: Japan, Australia, Germany, Sweden, the Netherlands, Spain, France, Italy, Denmark, Greece, Switzerland, Israel, Belgium, Czech Republic, Poland, and Ireland. Both surveys collected information on the sociodemographic characteristics and health circumstances of representative adults aged 50 and older (including comparable measurements of cognitive impairments for the cross-sectional waves used in analysis^2^). Given the baseline survey of JSTAR only consists of individuals aged 50–75, we restricted our sample to non-institutionalized people aged 50–75 in both datasets.

For the first objective, to assess the (differential) occurrence and scope of wealth-based health inequalities among older adults, we performed our analysis at the country level. For the second objective, we pooled the two surveys, producing 16 countries with 31,969 individuals for physical impairments and 31,348 individuals for cognitive impairments after dropping all missing values.

### Measures

#### Physical impairments

We used two common measures of physical health, limitations in activities of daily living (ADL) and instrumental activities of daily living (IADL), to define physical impairments. Specifically, the ADL domain comprised six tasks: dressing, walking, bathing, eating, getting in or out of bed, and using the toilet. The IADL domain consisted of seven instrumental activities: using a map, preparing a hot meal, shopping, using a telephone, taking medications, doing housework or garden work, and managing money. We created a score of physical impairments, reflecting the sum of all ADL and IADL, ranging from 0 to 13. Higher scores indicated a higher level of physical impairments.

#### Cognitive impairments

This study focused on fluid cognitive skills such as memory and recall, which refer to learning new tasks and remembering and processing new information. While these cognitive skills have been found to be negatively affected by cognitive aging, crystallized cognitive skills such as numerical and verbal skills reflect accumulated experience and wisdom and are relatively stable in old age [43]. Furthermore, the distributions of recall scores are normally distributed and do not suffer from floor or ceiling effects [44]. We measured cognitive impairments based on immediate and delayed recalls. The immediate recall measured how many of 10 words the respondent could recall immediately after the interviewer read the words. After a while, the delayed recall was conducted, and it repeated a similar procedure to the immediate one. The sum of the immediate and delayed recalls represented the cognitive impairments score. To ease interpretation and comparison with physical impairments, we applied reverse coding such that a higher cognitive impairments score indicated worse cognitive function. Since the immediate and delayed recalls used the same word list, this may raise a concern of “learning effects” due to repeated exposure to the same test, which tend to increase the test scores of some respondents [45]. To examine the potential bias caused by learning effects, we used only the immediate recall to measure cognitive impairments in a robustness check.

#### SES measure

As our SES measure, we used household wealth, defined as the sum of net real and net financial assets minus debts. Financial assets represented the sum of the values of accounts, bonds, stocks, mutual funds, and savings. Real assets consisted of the value of the primary residence net of mortgage, other real estate, owned businesses, and owned cars. Following previous studies, we applied the inverse hyperbolic sine transformation in the household wealth variable to address the skewed distribution and zero- and negative-valued observations to capture debt, for instance. The coefficients of arcsin-transformed variables can be interpreted similarly to the coefficients of log-transformed variables [46, 47].

Since JSTAR only provides information on respondents and their spouses, household wealth was measured by the contribution of either (i) both the respondents and their spouses if they managed their household finances together or (ii) the respondent solely if they managed their household finances separately. However, SHARE contains information on other household members in addition to the respondents and their spouses. To make the two datasets comparable, we converted wealth in JSTAR into Euros using the average annual exchange rates for the respective years^3^ and limited our analytic sample to households with a maximum of two people. We conducted a robustness check using household wealth per capita to account for the number of household members as the measure of SES. We then repeated all the analysis procedures on the SHARE data, excluding JSTAR. Results of this robustness check are presented in Online Appendix, showing that our main results are robust to the alternative measure of household wealth.

#### Country-level independent variables

Given our interest in the association between country-level (i.e., macro) factors and wealth-impairment inequalities, we considered four macro characteristics that reflect healthcare spending and infrastructure. All the macro measures were drawn from the Organization for Economic Cooperation and Development (OECD) database for 2007, thereby aligning with the survey years for the individual-level data [48]. We used the share of public health spending^4^ and share of OOP expenditure in each country, each measured compared with total health spending, expressed as a percentage of GDP. Notably, each measure was compared across rather than within countries. For instance, we compared public and OOP health expenditure in Japan with that in Belgium as opposed to comparing those metrics within each country. For healthcare infrastructure, we used the number of doctors and hospital beds per 1,000 inhabitants. According to the OECD, the number of hospital beds comprise beds from both public and private hospitals including rehabilitative care beds, long-term care beds, and other beds.

### Statistical analysis

#### Concentration index (CI)

We utilized a CI to measure the degree of wealth-related inequalities in physical and cognitive impairments, expressed as follows:

1 $$\mathrm{CI}=\frac2{N\mu}{\textstyle\sum_{i=1}^N}\;{\mathrm w}_{\mathrm i}{\mathrm y}_{\mathrm i}{\mathrm R}_{\mathrm i}-1$$

where *N* is the sample size; ${y}_{i}$ is the health variable of interest of individual *i* (physical or cognitive impairments) and *µ* is its weighted mean; ${w}_{i}$ is the personal weight provided by the harmonized JSTAR and SHARE; and ${R}_{i}$ is the weighted fractional rank of the *i*^th^ individual in the wealth distribution [1].

The CI has several advantages as a measure of wealth-related inequalities in health. Most importantly, it reflects the experience of the entire population and not just those of two extreme socioeconomic groups. However, it does not account for the health of the population. Since the average health of the population differs by country, to make the CI comparable, we computed a generalized CI (hereafter referred to as the CI for simplicity) based on the mean of the impairment outcomes in each country [4].

We further used an indirect method to standardize the CI by age and sex, controlling for the respondents’ educational level, as physical and cognitive health varies by age, sex, and education [49, 50]. Controlling for education helped minimizing omitted variable bias when standardizing [51]. In this analysis, to avoid having a small proportion of the sample within multiple age groups, we categorized age into two groups (e.g., 50–64 and 65–75) and interacted these with sex, yielding four age-sex groups. We measured educational level according to the International Standard Classification of Education (ISCED 97) with three categories: less than upper secondary school (reference), upper secondary, and vocational training and tertiary education. Detailed information on the indirect method standardization for CI has been presented elsewhere [51].

Both the standardized CIs and CIs ranged from -1 to + 1, with -1 indicating a disproportionate concentration of the impairments among the less wealthy (negative association between wealth and impairments), 0 indicating no socioeconomic inequality in the distribution of impairments, and + 1 indicating a disproportionate concentration of the impairments among the wealthiest (positive association between wealth and impairments). The online Appendix Table A1 presents the standardized and CIs for both impairments.^5^ Although the CIs for physical and cognitive impairments were similar to the standardized CIs, the magnitude of the inequality in most countries was slightly higher. Thus, to simplify the presentation of the results, we present only the standardized CIs hereafter. To facilitate interpretation, a lower negative CI indicates a larger magnitude of inequality among the least wealthy than a higher negative CI (e.g., CI -0.207 vs. -0.03), and vice versa for positive CIs.

#### Association between health inequalities in impairments and macro determinants

We first examined the correlation between the standardized CI and each macro indicator. Next, we examined the relationship between health inequalities and the macro determinants by applying multilevel linear regression models to account for the interdependence of the individuals nested within the studied countries. We used a random intercept model, with individuals and countries constituting levels 1 and 2, respectively, as follows:

2 $${\mathrm y}_{\mathrm{ij}}=\beta_0+\beta_1{\mathrm W}_{\mathrm{ij}}+\beta_2{\mathrm M}_{\mathrm j}+\beta_3{\mathrm W}_{\mathrm{ij}}\;\ast\;{\mathrm M}_{\mathrm j}+\mu_{0\mathrm j}+\mu_{1\mathrm j}{\mathrm W}_{\mathrm{ij}}+\varepsilon_{\mathrm{ij}}$$

where ${y}_{ij}$ is the outcome of individual *i* in country *j*; and stand for the wealth and macro-level factors, respectively; is the random residual term at the country level, which is assumed to have a mean of zero and be independent of the residual term at the individual level${\varepsilon }_{ij}$; and ${\mathrm{u}}_{1j}$ is the random error term at the country level. We are interested in ${\beta }_{3}$ ,which is the cross-level interactions between individual-level wealth (level 1) and the macro-level factors (level 2), as this captures the impacts of the public health spending and healthcare access macro indicators on the association between wealth and physical and cognitive impairments.

## Results

### Descriptive statistics

Table 1 summarizes the means of physical and cognitive impairments across the 16 countries in our study. Israel and Italy respectively had the highest levels of physical and cognitive impairments on average, whereas Switzerland and Denmark respectively reported the lowest average levels. Online Appendix Tables A2 and A3 provide the descriptive statistics for the mean wealth and values of the macro indicators across the countries. In general, these statistics varied considerably by country.

**Table 1:** Mean and standard deviation of physical and cognitive impairments by country

	No. of observations	Physical impairments	No. of observations	Cognitive impairments
Japan	3,312	0.311(1.219)	2,988	10.682(3.334)
Austria	805	0.362(1.275)	802	10.031(3.505)
Germany	2,158	0.271(1.096)	2,134	10.241(3.049)
Sweden	2,189	0.249(1.003)	2,175	9.779(3.059)
Netherlands	2,234	0.272(1.079)	2,212	10.097(3.359)
Spain	1,627	0.341(1.313)	1,612	13.021(3.154)
Italy	2,380	0.347(1.288)	2,363	11.835(3.494)
France	2,242	0.272(1.033)	2,189	11.367(3.320)
Denmark	2,078	0.264(1.086)	2,070	9.480(3.179)
Greece	2,513	0.286(1.091)	2,499	11.387(2.960)
Switzerland	1,138	0.150(0.779)	1,134	10.061(3.176)
Belgium	2,480	0.360(1.173)	2,464	10.794(3.316)
Israel	1,747	0.782(1.899)	1,689	11.533(3.295)
Czech Republic	2,218	0.278(1.013)	2,195	11.028(3.077)
Poland	2,018	0.772(1.825)	2,008	12.409(3.229)
Ireland	830	0.389(1.238)	814	10.273(3.674)
Total	31,969		31,348	

Standard deviations are in parentheses

Physical and cognitive impairments varied by age, sex, and educational level (Online Appendix Figures A1 to A3), suggesting the need to account for these characteristics when measuring socioeconomic inequalities in such impairments.

### Wealth inequalities in impairments

**Fig. 1:**
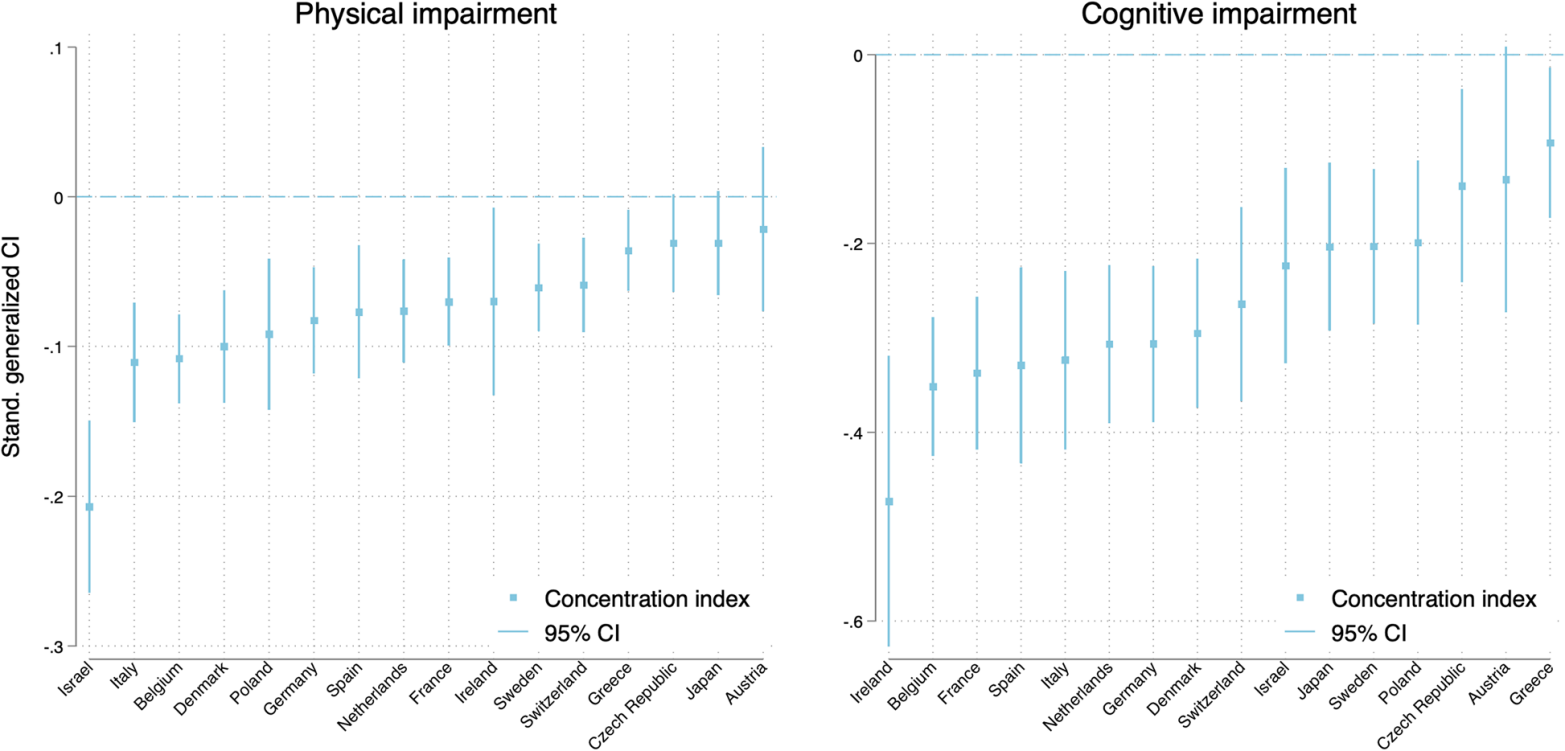
Standardized CIs with 95% confidence intervals for physical and cognitive impairments

Figure 1 presents the standardized CIs by country.^6^ Although all the standardized CIs were negative, suggesting that impairments were concentrated among the least wealthy, we found negative and statistically significant values for cognitive impairments in all 16 countries except Austria and for physical impairments in 13 countries (all 16 countries except Japan, Austria, and Czech Republic). Our analyses also indicated that wealth inequality varied between the countries according to the impairment outcomes. Specifically, Greece had the lowest wealth inequality for physical impairments (-0.036), whereas Israel had the highest (-0.207). Regarding cognitive impairments, Greece also showed the lowest wealth inequality (-0.093), whereas Ireland showed the highest (-0.473).

**Fig. 2:**
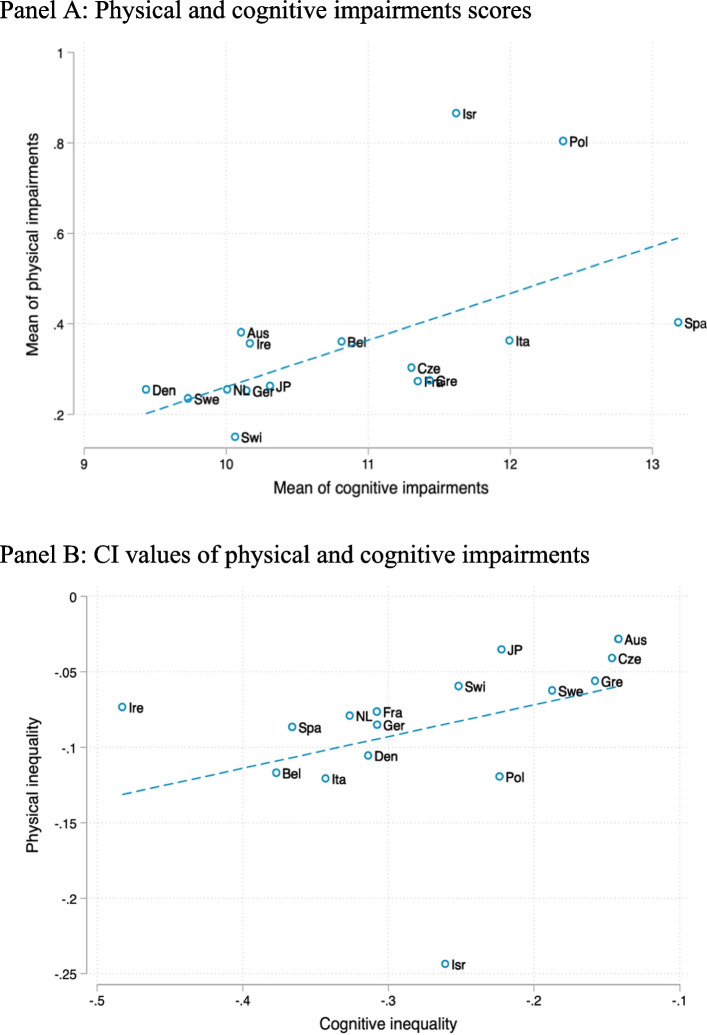
Correlations between physical and cognitive impairments. Panel **A**: Physical and cognitive impairments scores, Panel **B**: CI values of physical and cognitive impairments. Note: Aus, Bel, Cez, Den, Fra, Ger, Gre, Ire, Irs, Ita, JP, NL, Pol, Spa, Swe, and Swi denote Austria, Belgium, Czech Republic, Denmark, Germany, Greece, Ireland, Israel, Italy, Japan, Netherlands, Poland, Spain, Sweden, and Switzerland

Although physical and cognitive impairments were positively correlated (Fig. 2), the magnitude of inequalities for cognitive impairments consistently exceeded those of physical impairments across the countries (Fig. 1), suggesting that individuals’ position in the wealth distribution within their countries had a stronger association with cognitive than physical impairments.

### Correlation between wealth inequalities in physical and cognitive impairments and macro indicators

Table 2 presents the correlations between the standardized CIs for both impairments and the macro-factors. The positive correlation between the standardized CIs (all negative values) and macro indicators (all positive values) suggests that an increase in the given macro indicator is associated with an increased standardized CI. Given the CI ranges from -1 to + 1, an increased standardized CI indicates movement towards 0 (equality), therefore positive correlations indicate smaller inequalities in the health outcomes. For physical impairments, higher OOP expenditure was correlated with higher inequalities, whereas higher public health expenditure as well as the greater availability of doctors and hospital beds was correlated with smaller inequalities. For cognitive impairments, a higher availability of doctors and hospital beds was correlated with smaller inequalities. Interestingly, both higher public health spending and higher OPP were correlated with larger inequalities in cognitive impairments. It should be noted that as these are correlation analyses to explore the relationship between two variables (Fig. 2), whereas our formal regressions-multilevel analyses that follow, control for covariates.

**Table 2:** Correlation between wealth-related inequalities in physical and cognitive impairments and macro variables

	Physical impairments		Cognitive impairments	
	Standardized CI (1)	CI (2)	Standardized CI (3)	CI (4)
Share of public health spending	0.344^***^	0.435^***^	-0.209^***^	-0.098^***^
OOP payments	-0.081^***^	-0.146^***^	0.366^***^	0.264^***^
Number of doctors per 1,000 inhabitants	0.309^***^	0.339^***^	0.136^***^	0.164^***^
Number of hospital beds per 1,000 inhabitants	0.476^***^	0.464^***^	0.187^***^	0.232^***^
N	31,696		31,384	

Standardized CI means generalized CI standardizing for age and sex, controlling for respondents’ education level. CI refers to generalized CI without standardizing for age and sex

^*^p < 0.05; ^**^p < 0.01; ^***^p < 0.001

### Multilevel regression results

**Fig. 3:**
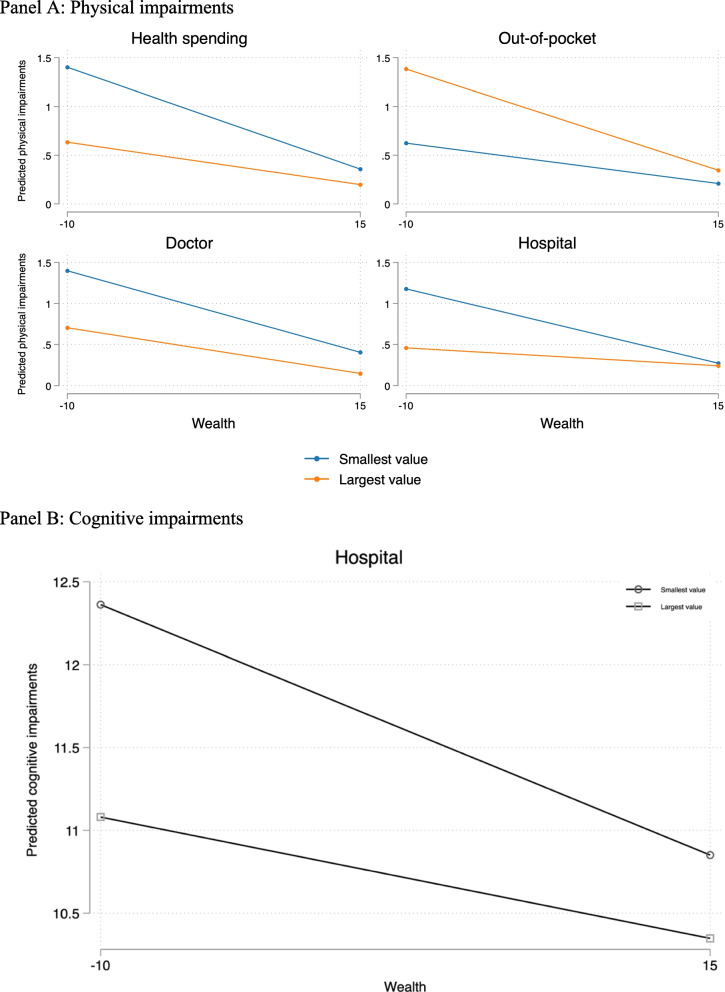
Predicted values of impairments at the lowest and highest values of the macro-level factors against the lowest and highest values of wealth. Panel **A**: Physical impairments. Panel **B**: Cognitive impairments. Notes: The graphs plot predicted values of physical and cognitive impairments (y-axis) at specific values of the macro-level factors (lowest and highest) against the lowest and highest values of wealth (x-axis). The circle and square markers indicate the lowest and highest values of the macro-level factors. Wealth is defined as the sum of net real and net financial assets minus debts, and thus contains negative values. Public health spending and OOP payments are measured compared with total health spending, expressed as a percentage of GDP. Doctor and Hospital refers to the number of doctors, and hospital beds (private and public), per 1,000 inhabitants, respectively

Table 3 shows the results of the multilevel analysis and Fig. 3 plots the predicted values of physical impairments (Panel A) and cognitive impairments (Panel B) using the lowest and highest values of each macro-level factor against the lowest and highest values of wealth. We only report the statistically significant coefficients of the interactions. We found that the differences in physical impairments by wealth were significantly smaller in countries with higher public health spending as well as more available doctors and hospital beds, suggesting that greater government investment in health is associated with lower wealth inequality (Panel A). Furthermore, in countries with lower OOP spending, we found significantly smaller wealth inequalities in physical impairments, suggesting that in countries in which individuals have to pay less toward their own healthcare, wealth differences are smaller. This implies that the adverse effect of lower wealth on health is mitigated by more generous healthcare resources. For cognitive impairments, only one macro indicator presented a statistically significant moderating effect (Panel B): wealth differences in cognitive impairments were lower in countries with more hospital beds. Overall, our findings suggest that the role of healthcare systems in mitigating wealth-impairment inequalities was more pronounced for physical than cognitive impairments.

**Table 3:** Cross-level interactions between household wealth and macro-level factors in their effect on impairment outcomes

Macro-level factor	Health spending Coef.(S.E.)	OOP payments Coef.(S.E.)	No. of doctors Coef.(S.E.)	No. of hospital beds Coef.(S.E.)
*Panel A: Physical impairments*	(1)	(2)	(3)	(4)
Wealth	-0.027^***^(0.002)	-0.026^***^(0.002)	-0.030^***^(0.002)	-0.028^***^(0.002)
Macro-level factor	-2.173^***^(0.557)	0.019^**^(0.006)	-0.226^**^(0.088)	0.038^*^(0.017)
Wealth × Macro-level factor	0.099^**^(0.019)	-0.001^***^(0.000)	-0.008^*^(0.004)	0.002^***^(0.000)
Intraclass correlation	0.016^***^(0.004)	0.020^**^(0.006)	0.016^*^(0.006)	0.019^**^(0.007)
Model fit (AIC)	103,610	104,662	93,755	104,603
N	31,696	31,696	31,696	31,696
*Panel B: Cognitive impairments*				
Wealth	-0.049^***^(0.004)	-0.049^***^(0.004)	-0.059^***^(0.004)	-0.050^***^(0.004)
Macro-level factor	-5.189(2.467)	0.041(0.028)	-0.623(0.402)	-0.088(0.077)
Wealth × Macro-level factor	0.035(0.050)	0.001(0.001)	0.012(0.009)	0.003^**^(0.001)
Intraclass correlation	0.055^**^(0.019)	0.058^***^(0.019)	0.063^**^(0.021)	0.066^**^(0.022)
Model fit (AIC)	158,210	159,665	143,507	159,660
N	31,348	31,348	31,348	31,348

All the models control for age-sex interactions and education; Coef. and S.E. denote coefficients and standard errors. The term “Macro-level factor” in the interaction term “Wealth × Macro-level factor” refers to each column in the first row when it crosses

^*^p < 0.05; ^**^p < 0.01; ^***^p < 0.001

### Robustness checks

We ran two robustness checks: using equivalized household wealth in the SHARE sample and using only the immediate recall to measure cognitive impairments. First, using equivalized household wealth, Online Appendix Figures A5(A) and A5(B) show that all the values of the standardized and CIs were negative. In addition, the magnitudes of inequality and the country order remained largely unchanged compared with the main results. The direction of the correlation between the CIs and macro determinants, presented in Online Appendix Table A4, was similar to that of the main results and these results held for both impairment outcomes. Compared with the main results, changes in the magnitude of the correlation were observed for both impairment outcomes, but the patterns of the changes were unclear. Moreover, the results of the multilevel regressions for both impairments, reported in Online Appendix Table A5, remained consistent with the main results. Second, the results of the immediate recall were similar to those of cognitive impairments (see Online Appendix Figure A6 and Appendix Tables A6 and A7). In sum, our estimates were unaffected by the measures of SES and learning effects.

## Discussion

Cross-country comparative studies on the association between health inequalities and macro determinants have provided many important insights, but little attention has been paid to health inequalities among middle-aged and older people. Furthermore, among these limited studies, household wealth, a crucial social economic resource in old age, is largely neglected. Since studies on SRH inequalities have dominated the literature, our study contributes to and advances our understanding of socioeconomic health inequalities in physical and cognitive impairments. We provide empirical evidence on wealth-impairment inequalities across Europe and Japan using comparable datasets as well as evidence on the extent to which macro indicators of healthcare financing and infrastructure mitigate the association between wealth and health in these countries.

Aligned with previous studies on the links among SRH, health limitations, and income [1, 17, 52, 53], and confirming our first hypothesis, our findings showed that physical and cognitive impairments were unequally distributed, favoring wealthier groups. Thus, poor health conditions are significantly concentrated on socioeconomically disadvantaged groups, even when using objective health measures. We observed both expected and unexpected results regarding the CI results. It is not surprising that Israel is a country with high wealth inequalities given the country’s low public health spending, high OOP and less investment on hospital beds (Appendix Table A3). It is, however, puzzling that Denmark shows relatively high wealth inequalities despite the high public health expenditure and investments in health care resources. This finding is consistent with those of some prior cross-country studies that show high health inequalities in countries with more egalitarian welfare systems, such as Denmark, Sweden, and Norway [1, 3, 17]. Previous studies have documented three reasons that explain this paradox in Nordic countries [54]. First, although welfare state helps redistribute income (thus, wealth) via taxation or other programs, inequalities in access to material living conditions persist. Second, intergenerational social mobility, which refers to cases where the next generation tend to have better social positions than previous ones due to better personal characteristics including cognitive ability, has been widespread in these countries [55]. One of the consequences of intergenerational social mobility being that socioeconomically advantaged individuals are likely selected into higher social positions thereby widening health inequalities as personal characteristics are closely related to health status. Finally, higher SES individuals may change their health behaviors earlier in their life course compared to their lower SES counterparts As a result, inequalities in health behaviors might be widening, as has been shown for countries with egalitarian welfare systems [54], and contributing to inequalities in cognitive and physical impairments as examined in the current study.

Considering the role of healthcare financing, we found differences in the effect of public health spending on the association between wealth and physical and cognitive impairments, partially supporting Hypothesis 2a. While higher public health spending was associated with lower wealth inequalities in physical impairments, no significant association was observed for cognitive impairments, which may be partially explained by the differences in the nature of these health dimensions. Specifically, physical impairments were measured using ADL and IADL, which could be diagnosed at early stages since physical functioning problems are (more) directly observable, especially among older people. If medical treatments are required for those with physical impairments, higher public health spending is expected to be associated with lower wealth inequalities, as proposed in our second hypothesis. By contrast, cognitive impairments may remain underdiagnosed by many people [56, 57], who may not seek healthcare, possibly masking the effects of public health spending on the association between wealth and cognitive impairments. Nevertheless, our findings for wealth inequalities in physical impairments are similar to those of studies that suggest that higher health expenditure is associated with lower health inequalities for subjective measures such as SRH [17, 18, 58]. Using an objective and a quasi-objective measure of health, our findings suggest that health is multifaceted and that its different dimensions should be investigated to better understand the association between macro-level factors and health inequalities.

Regarding the association between wealth-impairment inequalities and OOP payments (Hypothesis 2b), we found that higher OOP expenditure was correlated with higher wealth inequalities in both impairments, although we did not find such a significant association in the multilevel regression results for cognitive impairments. Higher OOP payments can result in catastrophic health expenditure when they exceed a certain threshold of household income. Such catastrophic health expenditure is more prevalent among vulnerable groups such as low-income and older people [16]. This might widen socioeconomic disparities between groups and health disparities in both impairments. Moreover, a high share of OOP expenditure has been shown to be a barrier to healthcare access in Europe [16]. Health inequalities (proxied by unmet health needs) disproportionately affect poor households due to financial barriers and this situation worsens in countries with a higher share of OOP payments [16, 59].

Our findings also partially support our third hypothesis on public healthcare resources. We found that the higher availability of doctors and hospital beds was associated with lower wealth inequalities in physical impairments. Indeed, expanding the availability of medical human resources and healthcare facilities principally aims to provide access to healthcare to people in need. Assuming that socially disadvantaged groups are the main targets of public policy, higher investment in healthcare access may reduce social inequalities in health, which may account for our finding of a relationship between the number of doctors/hospital beds and wealth inequalities in physical impairments. For cognitive impairments, there was no statistically significant moderating effect for the number of doctors unlike the number of hospital beds. This finding likely reflects the limited healthcare utilization among individuals with cognitive impairments. By contrast, the number of hospital beds may reflect hospital capacity and quality of care for severe impairments, as this indicator consisted of both rehabilitative care and long-term care beds, thus providing a sense of security for cognitively impaired older adults.

We acknowledge the limitations of this study. First, as a cross-sectional comparative study, it could not derive causal interpretations or provide mechanisms that explain our findings. Second, another implication of using cross-sectional data is that we could not account for attrition due to mortality. If mortality is systematically related to individuals’ wealth, it may affect the relationship between wealth and health observed in our study. Finally, although physical impairments are considered as a quasi-objective measure of health, reporting bias may still exist. This could be culturally sensitive since people may not report their physical difficulties in the same way.

Despite these limitations, our study has several strengths that provide a baseline for future studies. First, we used harmonized datasets across European countries and Japan to produce comparable estimates and future studies could enhance our research with additional global harmonized health measures. Additionally, our findings provide strong empirical evidence on wealth inequalities not only in physical impairments but also in cognitive impairments, favoring wealthier older adults in advanced aging societies. As societies age and healthcare demands increase, it is increasingly important for societies to identify the determinants of health inequalities and complementary social policy actions. Our findings suggest that health is multidimensional; hence, different health policy and interventions may be needed to mitigate wealth inequalities in certain impairments. Specifically, greater investment in healthcare resources, a higher share of public health spending, and a lower share of OOP expenditure are more strongly associated with lower wealth inequalities in physical impairments, whereas greater investment in hospital capacity to meet the needs of cognitively impaired older adults may be more critical to mitigating wealth inequalities. Thus, health policies indeed present intervention possibilities to secure healthy aging for all in aging societies globally, but they must be tailored to specific health outcomes.

## Supplementary Information

**Additional file 1: Table A1.** Unstandardized and standardized Cis. **Table A2.** Mean and standard deviation of wealth by country^a^. **Table A3.** Macro-level factors in 2007 by country^a^. **Table A4.** Robustness check using equivalized household wealth and excluding Japan. **Table A5.** Cross-level interactions between equivalized household wealth and macro-level factors in their effect on impairment outcomes (excluding Japan). **Table A6.** Robustness check using the immediate recall. **Table A7.** Cross-level interactions between household wealth and macro-level factors in their effect on immediate recall. **Figure A1.** Average physical and cognitive impairments scores with 95% confidence intervals by age. **Figure A2.** Average physical and cognitive impairments scores with 95% confidence intervals by age and sex. **Figure A3.** Average physical and cognitive impairments scores with 95% confidence intervals by age and educational level. **Figure A4.** CIs with 95% confidence intervals for physical and cognitive impairments. **Figure A5.** Results of the standardized CIs and CIs: Robustness check using equalized household wealth in the SHARE data. **Figure A6.** Results of the standardized CIs and CIs: Robustness check using only the immediate recall to proxy for cognitive impairments.

## Data Availability

We are not allowed to publicly share e datasets used in this study due to agreements with the dataset’s owner. The SHARE and JSTAR surveys data are available from the corresponding websites: http://www.share-project.org/home0.html and https://www.rieti.go.jp/en/projects/jstar/. Access to the data is limited to those who have registered for access and explained their intent to use the data.

## Footnotes

1 We use the Harmonized SHARE dataset and Codebook, Version E.2 as of November 2019 and the Harmonized JSTAR dataset and Codebook, Version B as of August 2014, developed by the Gateway to Global Aging Data. Additionally, for further methodological details on data from SHARE Wave 2 (DOIs: 10.6103/SHARE.w2.710) see Börsch-Supan et al. (2013).

2 In latter waves of SHARE (e.g., waves 4, 5 or latter) questions on immediate and delayed word recalls were repeated four times each, while in latter waves of JSTAR (e.g., waves 2, 3, or 4) immediate and delayed word recalls were repeated once in wave 2 or three times in waves 3. In addition, questions on immediate and delayed word recalls were not consistent for the fresh and follow-up samples in waves 3 and 4 of JSTAR. For example, in the refreshment sample, such questions were asked once with the same list of words for both immediate and delayed word recall. By contrast, in the follow-up sample, such questions were repeated three times and the lists of words for immediate recall were different from those of delayed recall. To maintain a comparable measure of cognitive impairment across countries we could not utilize data from later waves of both the SHARE and JSTAR and only cross-sectional analyses were performed.

3 We used exchange rates information from International Monetary Fund to convert Japanese Yen into Euros: https://www.imf.org/external/np/fin/data/rms_mth.aspx?SelectDate=2007-07-31&reportType=REP

4 Health spending measures the final consumption of healthcare goods and services including personal healthcare (curative care, rehabilitative care, long-term care and medical goods) and collective services (prevention and public health services as well as health administration) but excluding spending on investments.

5 In this Table, we tested whether standardized CIs (Columns 1 and 3) and CIs (Columns 2 and 4) are statistically significant different from zero or not.

6 The CIs are presented in Appendix Figure A4.
