# Supplementary material for: Wealth inequalities in physical and cognitive impairments across Japan and Europe: the role of health expenditure and infrastructure
Source: Int J Equity Health. 2023 Jun 29;22:123. doi: 10.1186/s12939-023-01906-6 (PMC10308628; doi:10.1186/s12939-023-01906-6)

**ONLINE APPENDIX TABLES AND FIGURES**

*Wealth inequalities in physical and cognitive impairments across Japan and Europe:*

*The role of health expenditure and infrastructure*

**TABLES**

Table A1: Unstandardized and standardized CIs

|  | Physical impairments | | Cognitive impairments | |
| --- | --- | --- | --- | --- |
|  | Standardized CI  (1) | CI  (2) | Standardized CI  (3) | CI  (4) |
| Japan | -0.031(0.018) | -0.035^*^(0.018) | -0.205^***^(0.045) | -0.224^***^(0.046) |
| Austria | -0.022(0.028) | -0.028(0.028) | -0.132(0.072) | -0.142(0.073) |
| Germany | -0.082^***^(0.018) | -0.085^***^(0.018) | -0.306^***^(0.042) | -0.308^***^(0.043) |
| Sweden | -0.061^***^(0.015) | -0.062^***^(0.015) | -0.203^**^(0.042) | -0.188^***^(0.044) |
| Netherlands | -0.071^***^(0.018) | -0.079^***^(0.018) | -0.307^***^(0.043) | -0.327^***^(0.044) |
| Spain | -0.077^***^(0.023) | -0.086^***^(0.023) | -0.329^***^(0.053) | -0.366^***^(0.055) |
| Italy | -0.111^***^(0.020) | -0.121^***^(0.020) | -0.324^***^(0.048) | -0.343^***^(0.049) |
| France | -0.070^***^(0.015) | -0.076^***^(0.015) | -0.337^***^(0.041) | -0.308^***^(0.043) |
| Denmark | 0.100^***^(0.019) | -0.105^***^(0.019) | -0.295^***^(0.040) | -0.312^***^(0.042) |
| Greece | -0.036^***^(0.014) | -0.056^***^(0.014) | -0.093^**^(0.041) | -0.158^***^(0.042) |
| Switzerland | -0.059^***^(0.016) | -0.059^***^(0.016) | -0.264^***^(0.052) | -0.252^***^(0.055) |
| Belgium | -0.108^***^(0.015) | -0.117^***^(0.015) | -0.352^***^(0.037) | -0.377^***^(0.039) |
| Israel | -0.207^***^(0.029) | -0.243^***^(0.030) | -0.224^***^(0.053) | -0.261^***^(0.053) |
| Czech Republic | -0.031(0.017) | -0.041^*^(0.017) | -0.139^***^(0.052) | -0.146^***^(0.054) |
| Poland | -0.092^***^(0.026) | -0.119^***^(0.027) | -0.199^***^(0.044) | -0.224^***^(0.045) |
| Ireland | -0.069^*^(0.032) | -0.073^*^(0.032) | -0.473^***^(0.078) | -0.483^***^(0.079) |
| N | 31,696 | | 31,348 | |

Note: Standardized CI means generalized CI standardizing for age and sex, whereas CI refers to generalized CI without standardizing for age and sex.

Robust standard errors are in parentheses.

^*^p < 0.05; ^**^p <0.01; ^***^p < 0.001.

Table A2: Mean and standard deviation of wealth by country^a^

|  | No. of observations | Wealth |
| --- | --- | --- |
| Japan | 3,312 | 9.895(6.083) |
| Austria | 805 | 11.511(3.410) |
| Germany | 2,158 | 11.281(4.443) |
| Sweden | 2,189 | 11.379(5.429) |
| Netherlands | 2,234 | 11.607(4.144) |
| Spain | 1,627 | 12.342(3.089) |
| Italy | 2,380 | 12.000(3.679) |
| France | 2,242 | 12.163(3.797) |
| Denmark | 2,078 | 12.008(4.741) |
| Greece | 2,513 | 11.776(3.422) |
| Switzerland | 1,138 | 11.926(4.924) |
| Belgium | 2,480 | 12.496(3.119) |
| Israel | 1,747 | 11.187(5.334) |
| Czech Republic | 2,218 | 10.377(3.465) |
| Poland | 2,018 | 9.006(4.705) |
| Ireland | 830 | 12.782(4.409) |
| N | 31,696 |  |

Note: Standard deviations are in parentheses. Household wealth is transformed using the inverse hyperbolic sine transformation and all country currency was converted to Euros.

^a^Since the means of wealth are similar for the physical and cognitive impairment samples across countries, for simplicity, we report the means of wealth according to physical impairments due to the larger number of observations.

Table A3: Macro-level factors in 2007 by country^a^

|  | N | Health spending | OOP payments | No. of doctors | No. of hospital beds |
| --- | --- | --- | --- | --- | --- |
| Japan | 3,312 | 81.2 | 15.5 | 2.1 | 13.9 |
| Austria | 805 | 74.4 | 18.8 | 4.5 | 7.8 |
| Germany | 2,158 | 74.9 | 14.3 | 3.5 | 8.2 |
| Sweden | 2,189 | 82.5 | 16.3 | 3.6 | 2.9 |
| Netherlands | 2,234 | 84.1 | 7.9 | 2.8 | 4.3 |
| Spain | 1,627 | 72.3 | 20.7 | 3.6 | 3.3 |
| Italy | 2,380 | 77.5 | 21.5 | 3.8 | 3.9 |
| France | 2,242 | 77.0 | 9.5 | 3.3 | 7.1 |
| Denmark | 2,078 | 83.7 | 14.6 | 3.5 | 3.7 |
| Greece | 2,513 | 63.3 | 34.2 | 3.7 | 4.9 |
| Switzerland | 1,138 | 62.4 | 27.3 | 3.8 | 5.4 |
| Belgium | 2,480 | 74.2 | 20.8 | 2.9 | 6.3 |
| Israel | 1,747 | 60.6 | 26.7 | 3.3 | 3.4 |
| Czech Republic | 2,218 | 84.7 | 13.6 | 3.6 | 7.3 |
| Poland | 2,018 | 70.1 | 26.3 | 2.2 | 6.4 |
| Ireland | 830 | 79.2 | 11.6 | 3.9 | 5.1 |
| N | 31,969 |  |  |  |  |

Note: Public health spending and OOP payments are measured compared with total health spending, expressed as a percentage of GDP. The numbers of doctors and hospital beds are measured per 1,000 inhabitants. All the macro-level factors are extracted from the OECD database in 2007: <https://data.oecd.org/healthres/health-spending.htm>.

^a^Similar to Table A1, the sample size is based on the physical impairments sample.

Table A4: Robustness check using equivalized household wealth and excluding Japan

|  | Physical impairments | | Cognitive impairments | |
| --- | --- | --- | --- | --- |
|  | Standardized CI  (1) | CI  (2) | Standardized CI  (3) | CI  (4) |
| Share of public health spending | 0.305^***^ | 0.379^***^ | -0.275^***^ | -0.209^***^ |
| OOP payments | -0.036^***^ | -0.083^***^ | 0.427^***^ | 0.379^***^ |
| Number of doctors per 1,000 inhabitants | 0.315^***^ | 0.335^***^ | 0.051^***^ | 0.093^***^ |
| Number of hospital beds per 1,000 inhabitants | 0.333^***^ | 0.320^***^ | 0.052^***^ | 0.119^***^ |
| N | 28,657 |  | 28,360 |  |

Note: Standardized CI means generalized CI standardizing for age and sex, whereas CI refers to generalized CI without standardizing for age and sex.

^*^p < 0.05; ^**^p <0.01; ^***^p < 0.001.

Table A5: Cross-level interactions between equivalized household wealth and macro-level factors in their effect on impairment outcomes (excluding Japan)

| Macro-level factor | Health spending  Coef.(S.E.) | OOP payments  Coef.(S.E.) | No. of doctors  Coef.(S.E.) | No. of hospital beds  Coef.(S.E.) |
| --- | --- | --- | --- | --- |
| *Panel A: Physical impairments* | (1) | (2) | (3) | (4) |
| Wealth | -0.031^***^(0.002) | -0.032^***^(0.002) | -0.031^***^(0.002) | -0.031^***^(0.002) |
| Macro | -1.778^**^(0.583) | 0.016^*^(0.017) | 0.227^*^(0.089) | -0.030(0.029) |
| Wealth ×Macro-level factor | 0.073^**^(0.022) | -0.001^**^(0.000) | 0.008^**^(0.004) | 0.002^*^(0.001) |
| Intraclass correlation | 0.016^**^(0.006) | 0.017^**^(0.006) | 0.016^**^(0.006) | 0.020^**^(0.007) |
| Model fit (AIC) | 93,470 | 93,411 | 93,413 | 93,417 |
| N | 28,675 | 28,675 | 28,675 | 28,675 |
| *Panel B: Cognitive impairments* |  |  |  |  |
| Wealth | -0.058^***^(0.004) | -0.057^***^(0.004) | -0.057^***^(0.004) | -0.059^***^(0.004) |
| Macro | -4.203(2.557) | 0.031(0.028) | 0.616(0.400) | 0.046(0.128) |
| Wealth ×Macro-level factor | -0.026(0.054) | 0.001(0.001) | 0.012(0.009) | 0.005^*^(0.002) |
| Intraclass correlation | 0.057^**^(0.019) | 0.059^**^(0.020) | 0.062^**^(0.022) | 0.069^**^(0.024) |
| Model fit (AIC) | 142,889 | 142,885 | 142,889 | 142,888 |
| N | 28,360 | 28,360 | 28,360 | 28,360 |

Note: all the models control for age-sex interactions and education; Coef. and S.E. denote coefficients and standard errors. The term “Macro” in the interaction term “Wealth ×Macro-level factor” refers to each column in the first row when it crosses.

^*^p < 0.05; ^**^p <0.01; ^***^p < 0.001.

Table A6: Robustness check using the immediate recall

|  | Immediate recall | |
| --- | --- | --- |
|  | Standardized CI  (1) | CI  (2) |
| Share of public health spending | -0.052^***^ | -0.080^***^ |
| OOP payments | 0.168^***^ | 0.042^***^ |
| Number of doctors per 1,000 inhabitants | 0.145^***^ | 0.148^***^ |
| Number of hospital beds per 1,000 inhabitants | 0.302^***^ | 0.356^***^ |
| N | 31,369 | |

Note: Standardized CI means generalized CI standardizing for age and sex controlling for respondents’ educational level. CI refers to generalized CI without standardizing for age and sex.

^*^p < 0.05; ^**^p <0.01; ^***^p < 0.001.

Table A7: Cross-level interactions between household wealth and macro-level factors in their effect on immediate recall

|  | Health spending  Coef.(S.E.) | OOP payments  Coef.(S.E.) | No. of doctors  Coef.(S.E.) | No. of hospital beds  Coef.(S.E.) |
| --- | --- | --- | --- | --- |
|  | (1) | (2) | (3) | (4) |
| Wealth | -0.026^***^(0.002) | -0.025^***^(0.002) | -0.028^***^(0.002) | -0.026^***^(0.002) |
| Macro | -1.993(1.194) | 0.016(0.013) | -0.258(0.195) | -0.027(0.037) |
| Wealth ×Macro-level factor | 0.000(0.025) | 0.001(0.001) | 0.004(0.005) | 0.0012*(0.0005) |
| Intraclass correlation | 0.050^**^(0.017) | 0.051^**^(0.017) | 0.057^**^(0.019) | 0.059^**^(0.019) |
| Model fit (AIC) | 115,989 | 115,987 | 104,982 | 115,986 |
| N | 31,369 | 31,369 | 31,369 | 31,369 |

Note: all the models control for age-sex interactions and education; Coef. and S.E. denote coefficients and standard errors. The term “Macro” in the interaction term “Wealth ×Macro-level factor” refers to each column in the first row when it crosses.

^*^p < 0.05; ^**^p <0.01; ^***^p < 0.001.

**FIGURES**

Figure A1 depicts the variation in physical and cognitive impairments by age, showing the negative association between physical and memory tests and age (i.e., the number of physical and cognitive impairments increased almost linearly with age).

Figure A1: Average physical and cognitive impairments scores with 95% confidence intervals by age


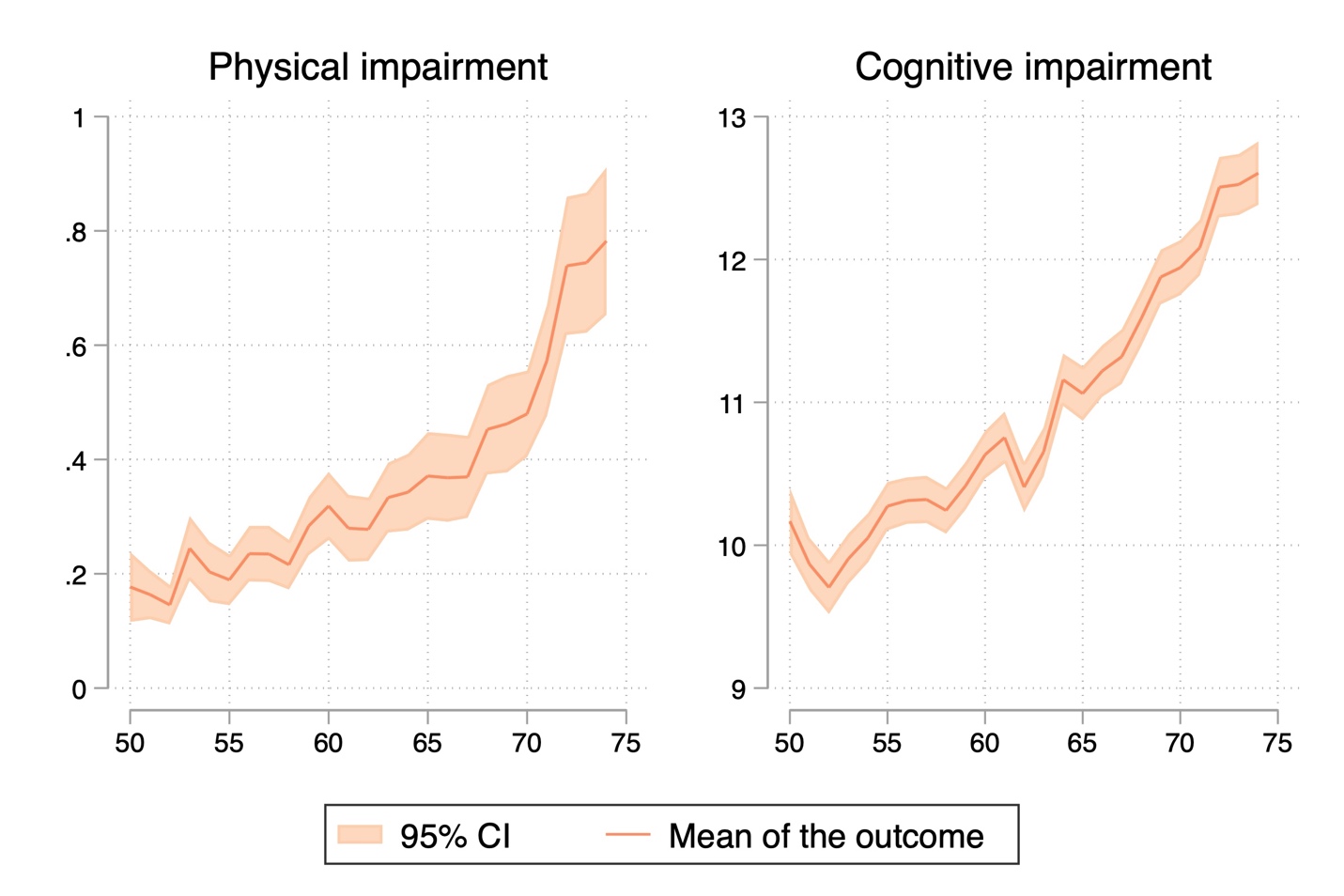


Figure A2 highlights the average of the two test scores differentiating for sex. Considerable sex differences between the two tests appear. Women scored worse than men at all ages in the physical tests, whereas they performed better across ages in the memory tests.

Figure A2: Average physical and cognitive impairments scores with 95% confidence intervals by age and sex


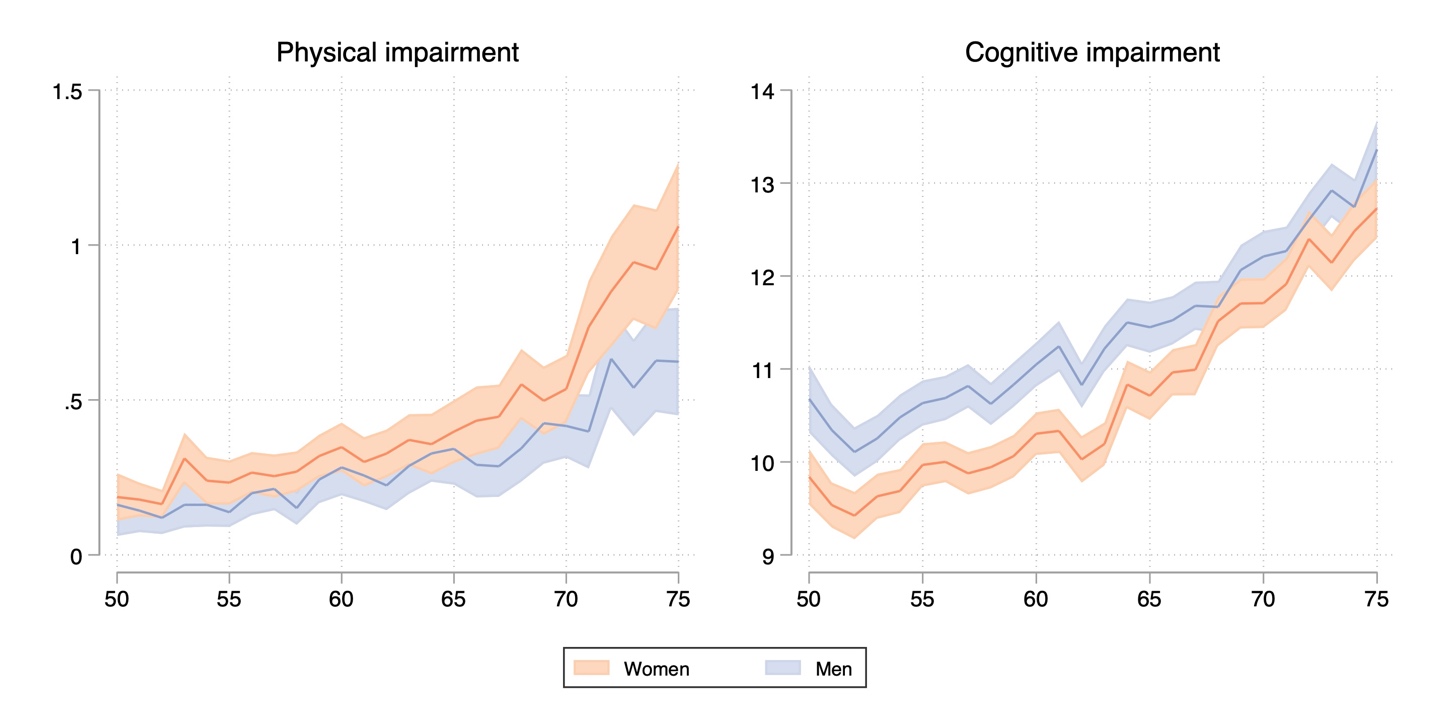


Figure A3 shows the average physical and cognitive impairments scores differentiating for educational level. Similar to Figure A2, differences in educational level vary considerably between the two tests. Although individuals with higher education tend to have lower physical impairments scores, differences in physical impairments scores across educational level are not pronounced. By contrast, educational level appears to play an important role in reducing cognitive impairments across all ages and educational levels. In sum, Figures A1 to A3 highlight the considerable variation in physical and cognitive impairments by age, sex, and education, thus indicating that these three important characteristics should be considered when measuring health inequalities.

Figure A3: Average physical and cognitive impairments scores with 95% confidence intervals by age and educational level


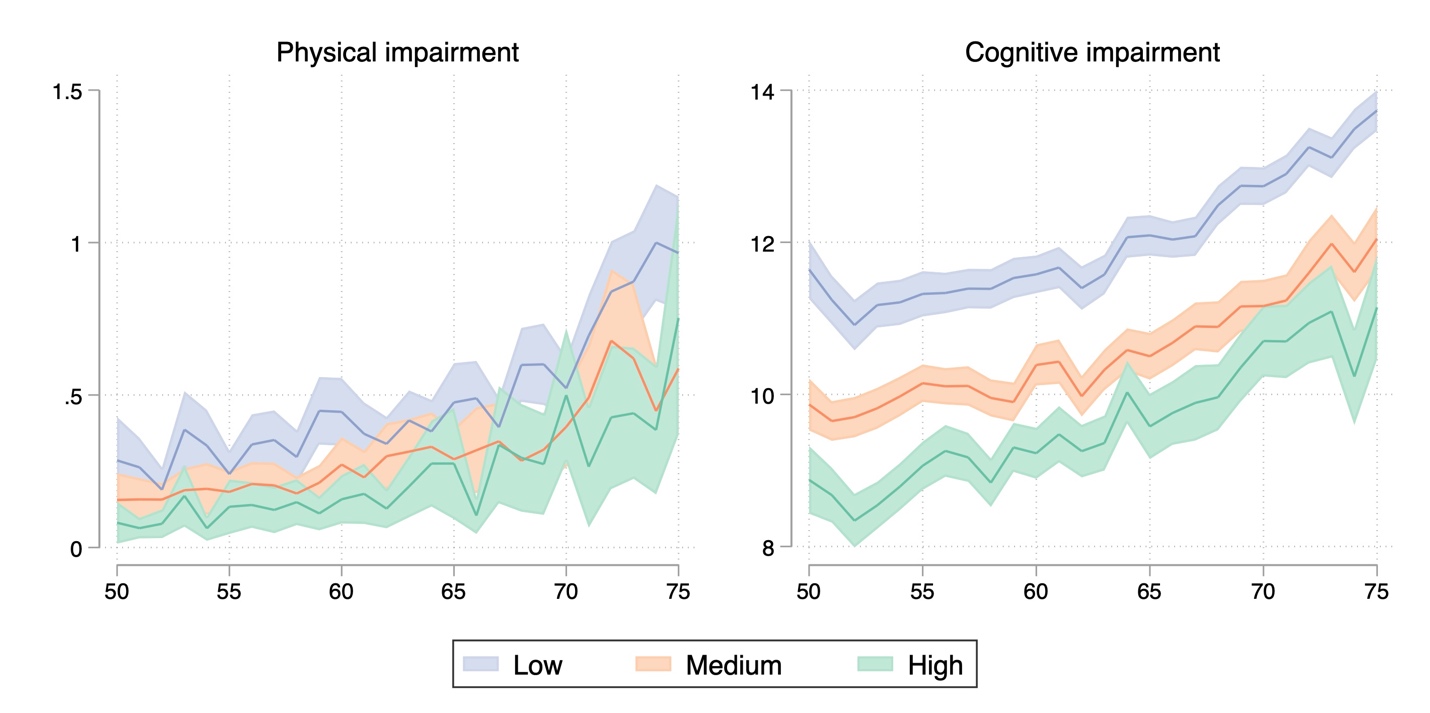


Figure A4: CIs with 95% confidence intervals for physical and cognitive impairments


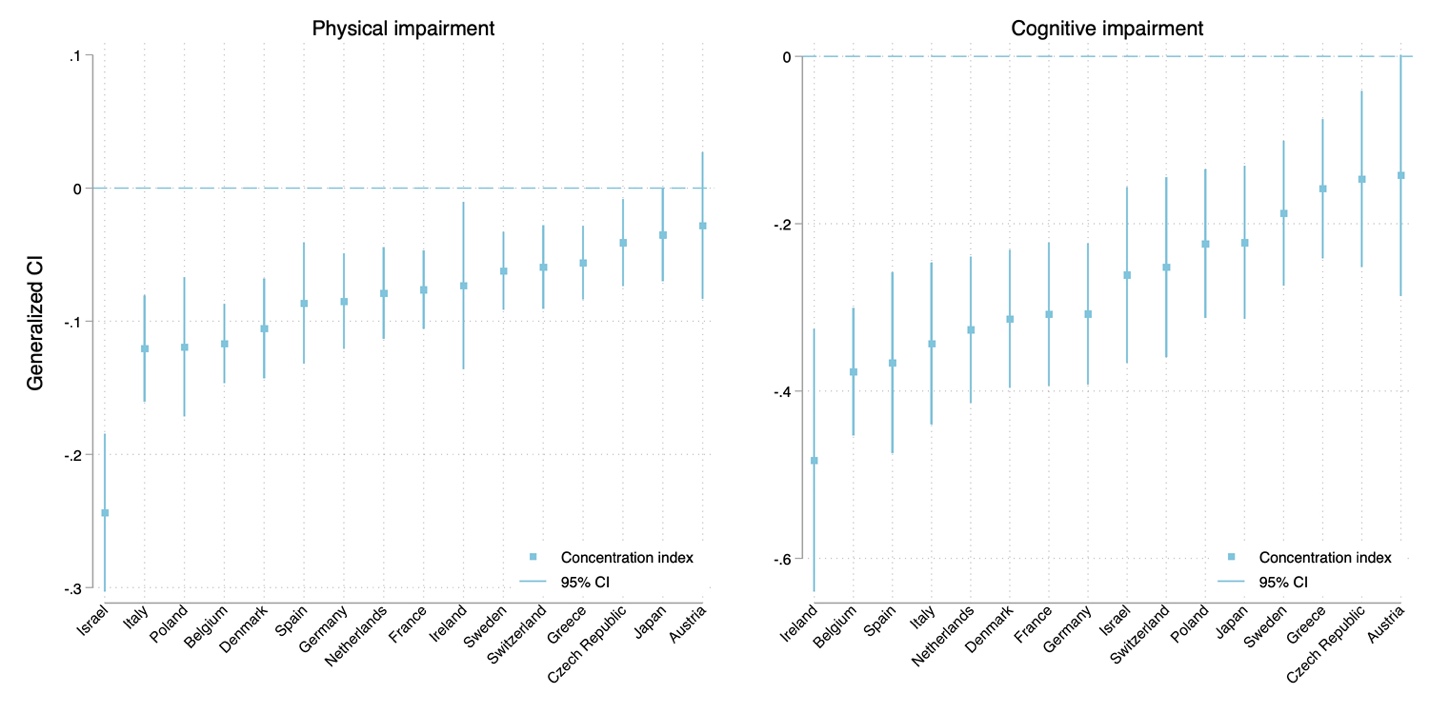


Figure A5: Results of the standardized CIs and CIs: Robustness check using equalized household wealth in the SHARE data

A: Standardized CIs


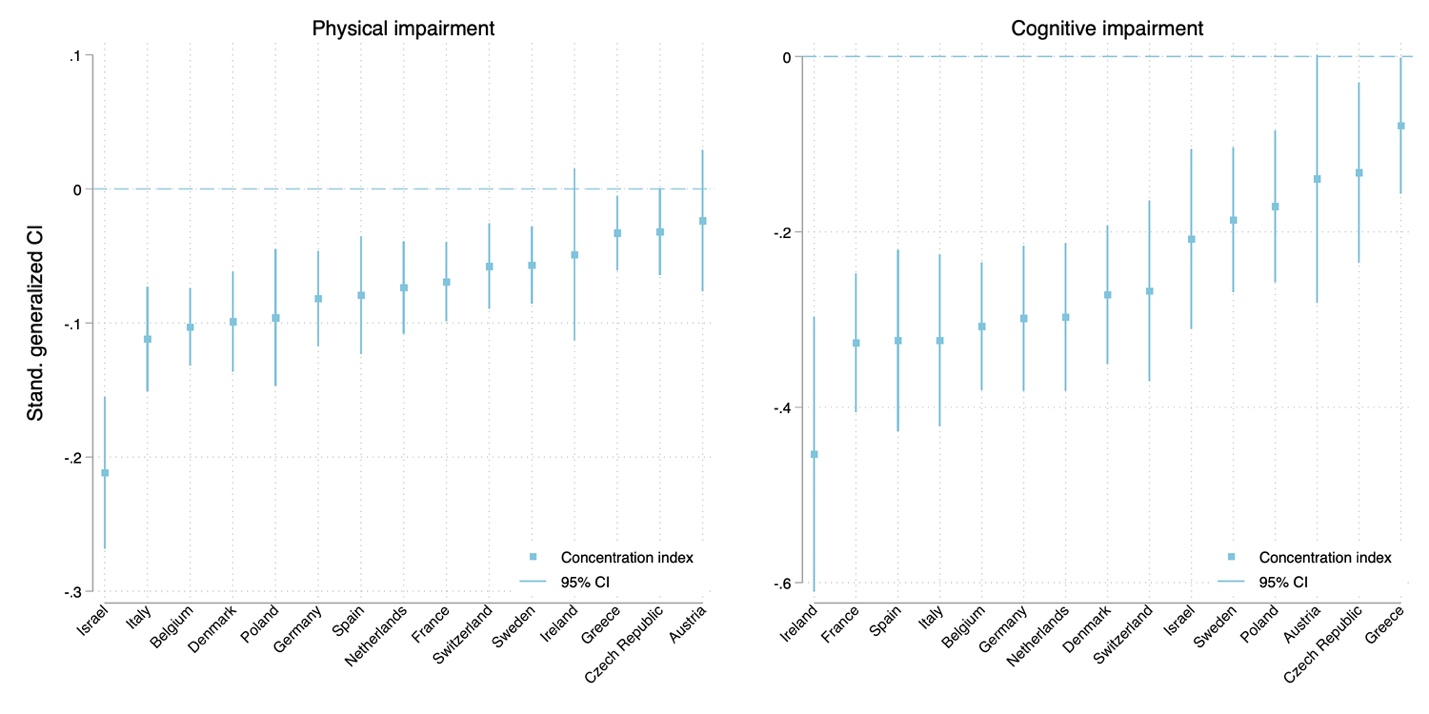


B: CIs


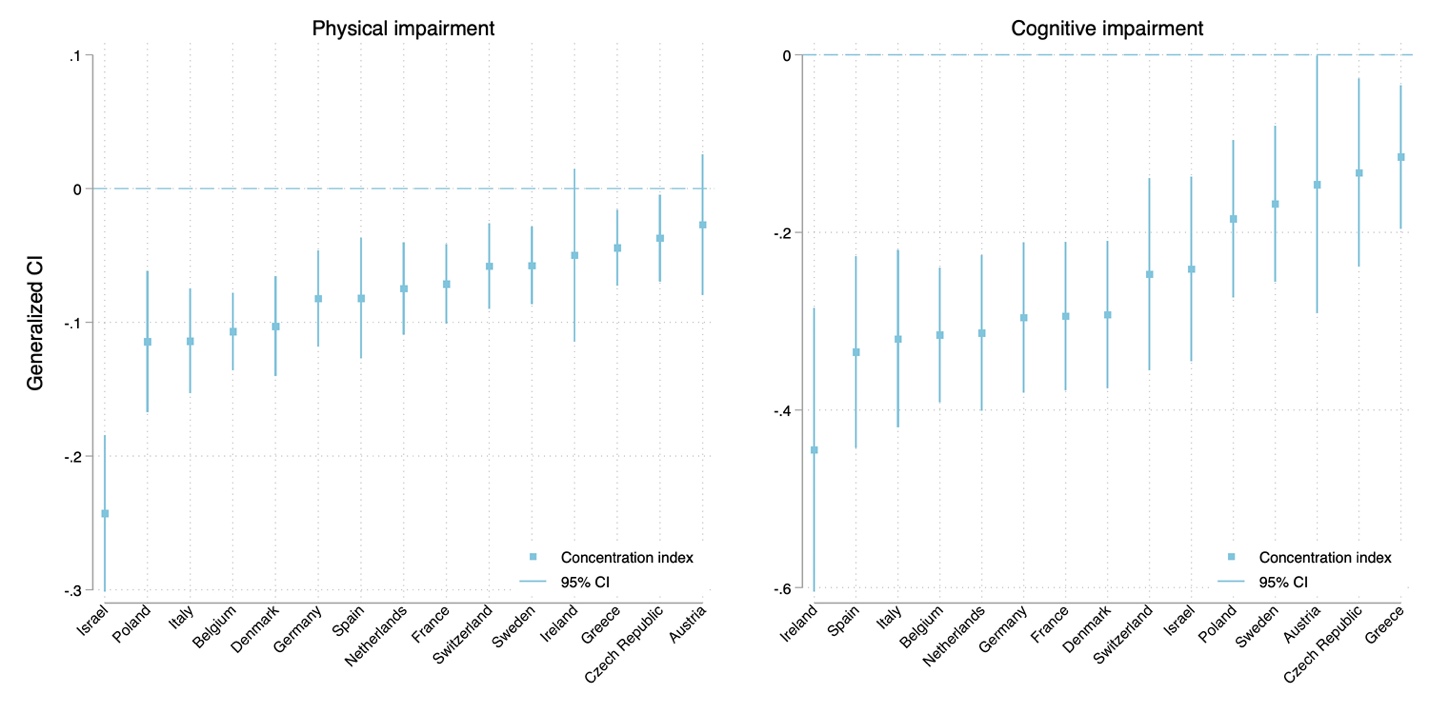


Figure A6: Results of the standardized CIs and CIs: Robustness check using only the immediate recall to proxy for cognitive impairments


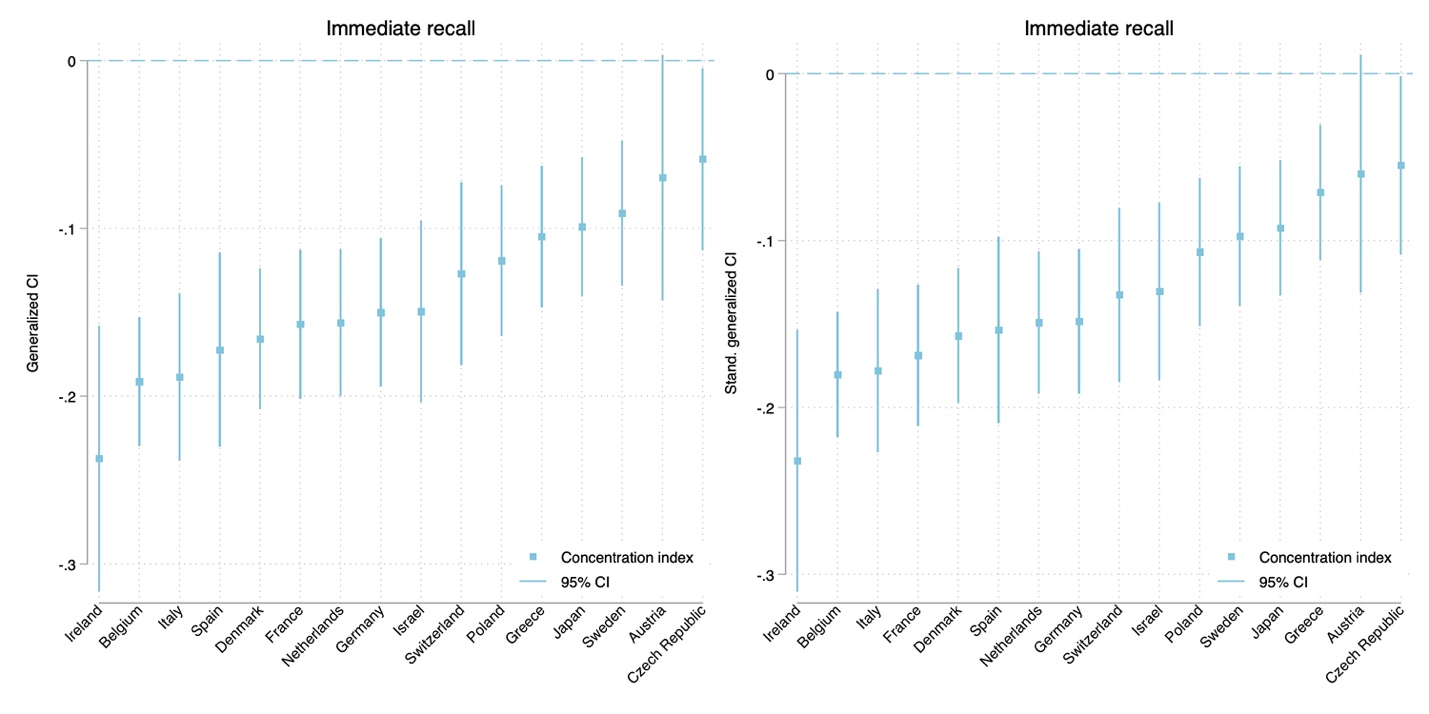

Supplement: Supplementary file 1 — Additional file 1: Table A1. Unstandardized and standardized Cis. Table A2. Mean and standard deviation of wealth by countrya. Table A3. Macro-level factors in 2007 by countrya. Table A4. Robustness check using equivalized household wealth and excluding Japan. Table A5. Cross-level interactions between equivalized household wealth and macro-level factors in their effect on impairment outcomes (excluding Japan). Table A6. Robustness check using the immediate recall. Table A7. Cross-level interactions between household wealth and macro-level factors in their effect on immediate recall. Figure A1. Average physical and cognitive impairments scores with 95% confidence intervals by age. Figure A2. Average physical and cognitive impairments scores with 95% confidence intervals by age and sex. Figure A3. Average physical and cognitive impairments scores with 95% confidence intervals by age and educational level. Figure A4. CIs with 95% confidence intervals for physical and cognitive impairments. Figure A5. Results of the standardized CIs and CIs: Robustness check using equalized household wealth in the SHARE data. Figure A6. Results of the standardized CIs and CIs: Robustness check using only the immediate recall to proxy for cognitive impairments. [file 12939_2023_1906_MOESM1_ESM.docx]
